# White matter abnormalities are key components of cerebrovascular disease impacting cognitive decline

**DOI:** 10.1093/braincomms/fcab076

**Published:** 2021-04-12

**Authors:** Prashanthi Vemuri, Jonathan Graff-Radford, Timothy G Lesnick, Scott A Przybelski, Robert I Reid, Ashritha L Reddy, Val J Lowe, Michelle M Mielke, Mary M Machulda, Ronald C Petersen, David S Knopman, Clifford R Jack

**Affiliations:** 1 Department of Radiology, Mayo Clinic, Rochester, MN 55905, USA; 2 Department of Neurology, Mayo Clinic, Rochester, MN 55905, USA; 3 Department of Quantitative Health Sciences, Mayo Clinic, Rochester, MN 55905, USA; 4 Department of Information Technology, Mayo Clinic, Rochester, MN 55905, USA; 5 Department of Psychiatry/Psychology, Mayo Clinic, Rochester, MN 55905, USA

**Keywords:** cerebrovascular disease, diffusion MRI, white matter hyperintensities, microbleeds, amyloidosis

## Abstract

While cerebrovascular disease can be observed *in vivo* using MRI, the multiplicity and heterogeneity in the mechanisms of cerebrovascular damage impede accounting for these measures in ageing and dementia studies. Our primary goal was to investigate the key sources of variability across MRI markers of cerebrovascular disease and evaluate their impact in comparison to amyloidosis on cognitive decline in a population-based sample. Our secondary goal was to evaluate the prognostic utility of a cerebrovascular summary measure from all markers. We included both visible lesions seen on MRI (white matter hyperintensities, cortical and subcortical infarctions, lobar and deep microbleeds) and early white matter damage due to systemic vascular health using diffusion changes in the genu of the corpus callosum. We identified 1089 individuals aged ≥60 years with concurrent amyloid-PET and MRI scans from the population-based Mayo Clinic Study of Aging. We divided these into discovery and validation datasets. Using the discovery dataset, we conducted principal component analyses and ascertained the main sources of variability in cerebrovascular disease markers. Using linear regression and mixed effect models, we evaluated the utility of these principal components and combinations of these components for the prediction of cognitive performance along with amyloidosis. Our main findings were (i) there were three primary sources of variability among the CVD measures—white matter changes are driven by white matter hyperintensities and diffusion changes; number of microbleeds (lobar and deep); and number of infarctions (cortical and subcortical); (ii) Components of white matter changes and microbleeds but not infarctions significantly predicted cognition trajectories in all domains with greater contributions from white matter; and (iii) The summary vascular score explained 3–5% of variability in baseline global cognition in comparison to 3–6% variability explained by amyloidosis. Across all cognitive domains, the vascular summary score had the least impact on memory performance (∼1%). Though there is mechanistic heterogeneity in the cerebrovascular disease markers measured on MRI, these changes can be grouped into three components and together explain variability in cognitive performance equivalent to the impact of amyloidosis on cognition. White matter changes represent dynamic ongoing damage, predicts future cognitive decline across all domains and diffusion measurements help capture white matter damage due to systemic vascular changes. Therefore, measuring and accounting for white matter changes using diffusion MRI and white matter hyperintensities along with microbleeds will allow us to capture vascular contributions to cognitive impairment and dementia.

## Graphical Abstract

**Graphical Abstract:**
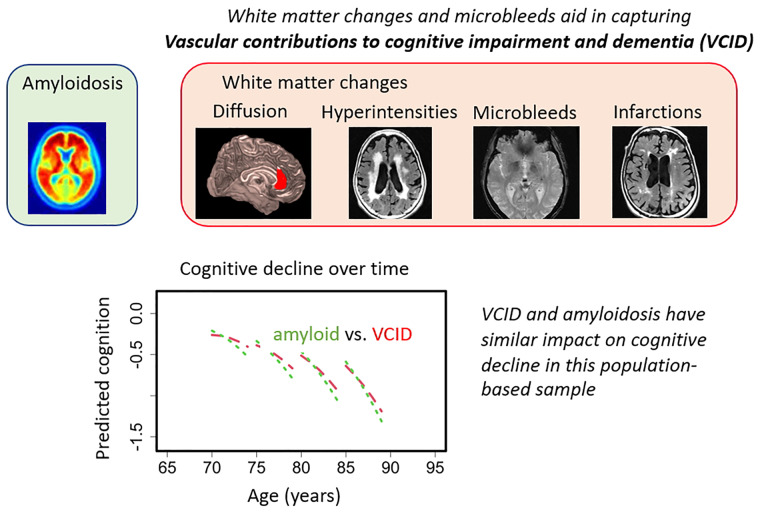

## Teaser

Vemuri et al. report that measuring and accounting for white matter changes (vascular health-related diffusion changes and white matter hyperintensities) and microbleeds will aid in capturing vascular contributions to cognitive impairment and dementia (VCID). VCID was equivalent to the impact of amyloidosis in this population-based sample of older adults.

## Introduction

Cerebrovascular disease (CVD) is common in the elderly and contributes to increased risk of cognitive impairment and dementia.^1^ CVD is a multifactorial process due to changes to the structure and function of the cerebral vasculature. This manifests in the form of blood–brain barrier dysfunction, impaired interstitial fluid drainage, cerebral blood flow alterations, white matter rarefaction, myelin damage and visible lesions such as white matter hyperintensities (WMHs), infarctions and microbleeds.^2^^,^^3^ The versatility of MRI allows us to capture a variety of mechanisms through which CVD impacts the brain.^4^ A recent systematic meta-analysis found that the presence and measurement of visible CVD on *in vivo* MRI has major clinical implications and is associated with an increased risk of dementia, stroke and death.^5^

Fluid-attenuated inversion recovery (FLAIR) and T_2_*-weighted gradient-echo (GRE) are the two MRI sequences that are most utilized to quantify CVD in ageing and dementia studies. These MRI sequences can detect the presence of microvascular changes [WMH and cerebral microbleeds (CMBs)] and macrovascular changes (subcortical and cortical macroinfarcts) in the brain. While there are several emerging neuroimaging technologies available to measure CVD and associated secondary neurodegeneration, diffusion MRI (dMRI) may be one of the most sensitive markers of early CVD changes because it can capture diffuse changes in normal appearing white matter (WM) even before the appearance of WMH^6^^,^^7^ and secondary loss of fibre tracts due to visible CVD. In a recent study, we showed that diffusion tensor imaging can capture subtle microstructural WM changes due to increased vascular risk even in the absence of visible CVD.^8^ Our study along with others^9^^,^^10^ support the utility of dMRI as a marker of CVD.

Each of these CVD changes individually and in combination would contribute to worse cognitive performance and lower the threshold for dementia.^11^ The multiplicity and significant heterogeneity in the CVD markers make it difficult to evaluate the prognostic value of CVD measures in ageing and dementia studies. We hypothesized that understanding the sources of variability across multiple CVD markers may allow us to summarize them effectively for clinical and research use. It will also allow us to understand how the individual CVD components impact the global and domain-specific cognitive processes. Alzheimer’s disease pathophysiology (amyloidosis) and CVD both contribute to the risk of cognitive impairment.^11^^,^^12^ Therefore, an important component of this work was to also compare the contribution of the CVD components to the predicting future decline in comparison to the contribution to amyloidosis. With this goal in mind, we aimed (i) to investigate the key sources/components of variability across the available surrogate MRI markers of CVD; (ii) to evaluate the clinical prognostic value of the CVD components along with amyloidosis; and (iii) to evaluate the utility of a summary score that can robustly capture all the available CVD information in a clinically useful measure. The present study included Mayo Clinic Study of Aging participants who had complete vascular assessments, dMRI, and amyloid imaging. We considered the following main components of CVD: FLAIR MRI for measurement of WMHs and infarctions (cortical and subcortical), T_2_* GRE for measurement of CMBs (lobar and deep), and dMRI for measurement of genu of the corpus callosum fractional anisotropy (FA GCC). We used amyloid-PET imaging using (11)C-labeled Pittsburgh Compound-B-PET as a surrogate for cerebral amyloidosis.^13^

## Materials and methods

### Selection of participants

The Mayo Clinic Study of Aging is a population-based study of Olmsted County, MN residents was enumerated using the Rochester Epidemiology Project medical records-linkage system.^14^^,^^15^ We included all 1089 individuals (ages ≥60 years) who had complete MRI vascular assessments on FLAIR, and dMRI, had concurrent amyloid imaging, and had at global and domain-specific cognitive testing at baseline and a follow-up visit. At the time of the scans, 107 were cognitively impaired [104 had mild cognitive impairment, 3 were diagnosed with a neurodegenerative disorder (1 with DLB, 1 with Parkinsonism and 1 with mixed dementia), and 2 had a missing clinical diagnosis due to incomplete data]. The Mayo Clinic Study of Aging design and clinical diagnoses criteria were discussed in detail by Petersen et al.^16^ and Roberts et al.^17^

#### Standard protocol approvals, registrations and patient consents

These studies were approved by the Mayo Clinic and Olmsted Medical Center institutional review boards. Informed consent was obtained from all participants or their surrogates.

### Demographic information

Sex, years of education and occupation information were obtained at the clinical visit. Age at the time of the MRI scan was considered. We computed education/occupation score as previously described^18^ and also ascertained the number of cardiovascular metabolic conditions (CMC) from health care records.^19^

### Cognitive performance

The neuropsychological battery consisted of nine tests covering four cognitive domains, as previously described.^16^^,^^17^ We used each of the individual *z*-scores as well as the average of the four-domain *z*-scores (attention/executive function, language; memory, and visuospatial performance) as cognitive outcomes.

### Imaging measures

**Figure 1:**
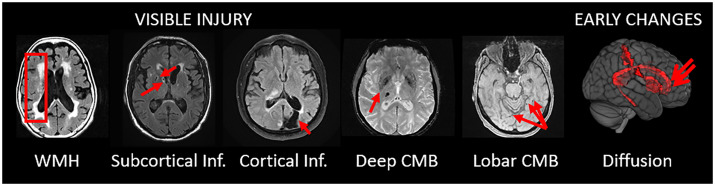
**Measured CVD-related changes on MRI**. The visible CVD injury/lesions are showed on the left and the tracts where early CVD related changes are measurable on diffusion MRI are shown to the far right.

All MRI images were acquired on 3 T MRI systems (GE Healthcare, Chicago, IL). The CVD image assessments on FLAIR, T_2_* GRE and dMRI were used in this study and shown in Fig. 1.

#### FLAIR and T_2_* GRE assessments

Briefly, all the CVD assessments on these images are explained here and the complete details of acquisition and processing can be found in Graff-Radford et al.^20^ WMH was segmented on standard two-dimensional T_2_ FLAIR using a semi-automated algorithm^21^ and edited by a trained analyst. We computed total intracranial volume using an SPM software based in-house implementation^22^ and used WMH as a percentage of total intracranial volume in this work. All possible CMBs (On T_2_* GRE) and infarctions (FLAIR) were marked by trained image analysts and subsequently confirmed by a vascular neurologist (JGR) experienced in reading T_2_* GRE and FLAIR scans and to whom the participants’ clinical information was masked. Cortical infarctions were characterized as hyperintense T_2_ FLAIR lesions (gliosis) involving cortical grey matter that extended to the cortical edge with or without involvement of the underlying WM (with confirmation from structural T_1_ images). Whereas, subcortical infarctions were characterized as hyperintense T_2_ FLAIR lesions with a dark centre, seen in the WM, infratentorial, and central grey-capsular regions. CMBs were defined based on current consensus criteria as homogeneous hypointense lesions in the grey or white matter that were distinct from iron or calcium deposits and vessel flow voids on the T_2_* GRE images.

#### dMRI assessment

The dMRI acquisition protocol was a 2.7 mm isotropic resolution spin-echo axial Echo Planar Imaging sequence with five *b* = 0 followed by 41 *b* = 1000 s/mm^2^ diffusion weighted volumes. We pre-processed data for Gibbs ringing,^23^ skull stripping, denoising,^24^ debiasing,^25^ head motion and eddy current distortion correction using FSL software, EPI distortion correction using BrainSuite.^26^ Diffusion tensors were then fit using nonlinear minimization after which Fractional Anisotropy and Mean Diffusivity were computed. ANTS software^27^ was used to nonlinearly register an in-house modified version of the JHU ‘Eve’ WM atlas^28^ to each participant’s FA image to compute regional median FA and MD. We recently found that integrity of the small fibres in the genu of the corpus callosum measured using dMRI based fractional anisotropy (FA GCC) captured both variability in systemic vascular health as well as visible cerebrovascular injury in the form of WMH.^8^ Therefore, we used that measure here as a marker of early CVD.

### Amyloid assessment

The acquisition and processing details of amyloid PET scans acquired on the study participants were discussed in detail by Jack et al.^29^ We computed the global amyloid load for each participant by calculating median uptake in the prefrontal, orbitofrontal, parietal, temporal, anterior cingulate and posterior cingulate/precuneus regions of interest divided by the median uptake in the cerebellar crus gray matter regions of interest.^29^

### Statistical methods

Standard summary measures were used to describe characteristics for all participants. We randomly divided the dataset into discovery (*n* = 544) and validation (*n* = 545) cohorts. The differences in participant characteristics (demographics, imaging measures and cognitive outcomes) between the discovery and validation cohorts were tested using *t*-tests for the continuous variables and chi-square tests for the categorical variables.

### Step 1: key sources/components of variability across MRI markers of CVD

Using the discovery dataset, we conducted principal component analyses (PCA) with the following assessments of CVD from T_2_* GRE, FLAIR, and FA GCC: WMH, FA GCC; continuous number of microbleeds, and continuous number of infarctions. The primary goals of the PCA were to reduce the dimensionality of the data, combine markers of underlying latent variables, and prevent multicollinearity problems from using correlated predictors in later analyses. WMH was log transformed to improve symmetry of the distribution. The assessments were centred and scaled before performing the PCA. We calculated the percent of variance explained by each component (of the total variance of all individuals PCs—principal components), ordered the components from greatest to least variance explained, and plotted the cumulative percent explained by one, two, three, etc. components. The final number of components was selected by choosing the number explaining at least 80% of the variance and showing some levelling on the cumulative curve. Following selection, we applied a varimax rotation to ease interpretation. The signs of the components were selected so that higher values would correspond to more CVD.

Our first PCA included deep and periventricular regional WMH, FA GCC, numbers of cortical and subcortical infarctions, and numbers of lobar and deep CMBs. In this study, the loadings for the WMH variables were almost equal, producing essentially a single sum for total WMH. The same was true for infarctions and CMBs. We accordingly reduced the data to single continuous variables for WMH, FA GCC, infarctions, and CMBs for the final PCA. This reduction makes sense for WMH, since regional WMHs are highly correlated^30^ and total WMH is the traditional cerebrovascular disease biomarker. Subcortical/cortical infarctions and lobar/deep CMBs might certainly have mechanistic differences, but the data driven reduction did not differentiate these variables.

### Step 2: prognostic value of the CVD components along with amyloidosis

Using components from the PCA analyses, we ran mixed effect models in the discovery data with random intercepts, slopes and curvature to examine the effects of the CVD components on longitudinal global and domain-specific *z*-scores (cognitive scores at baseline and follow-up visits were considered in the model). Age at baseline, time from baseline, time^2^ (quadratic term for curvature), sex, education/occupation, log(PiB), each of the PCs, and interactions of time with age, sex, education/occupation, log(PiB), and each of the PCs were the predictors. We tested for interactions of time^2^ with other predictors but did not find any to be significant. We also tested for within-subject autocorrelation using a continuous AR(1) correlation structure in those models, but again did not find significance. We started with all predictors in the each of the models, and then used backwards elimination to reduce these to parsimonious models. Finally, we applied the terms in the parsimonious models from the discovery data set to mixed models in the validation dataset. As a sensitivity check into possible differences in low versus high amyloid groups, we also performed all analyses in A− and A+ strata determined using an established cut point of 1.48 SUVR.^31^ We report the coefficients, their associated standard errors and *P*-values, and Nakagawa *R*^2 32,33^ for the fixed effects in each mixed model.

### Step 3: prognostic value of a summary vascular score

Because some of the CVD events are rare (CMBs and infarctions), our final goal was to evaluate if a summary score can robustly capture the available CVD information in a clinically useful measure. We investigated several ways of combining these measures and decided to create a summary measure using a simple summation of all PCs (retaining their scaling through PCA) because of the following two reasons: (i) conditioning on the outcome (i.e. estimating the weighting of each PC based on its impact on cognition) will yield a score sensitive to the population being investigated which reduces the generalizability of the scores; (ii) the *R*^2^ values for the vascular score models (with a simple summation) were similar to those for the more complicated PC models where each of the PCs were included. Therefore, goodness of fit was not reduced substantially by forming the summary vascular variable. Calculating the summary vascular score as a simple linear summation of PC1, PC2 and PC3, we estimated the contribution of the summary vascular score in cross-sectional linear regression models with baseline cognition as an outcome as well as mixed effect models with longitudinal cognition as an outcome using the discovery data set. Backwards elimination was used to form parsimonious models, and we applied the terms in the parsimonious models from the discovery data set to mixed models in the validation dataset. We again performed sensitivity analyses in the A− and A+ groups.

### Data availability

Data from this study are available from the authors upon reasonable request.

## Results

**Figure 2:**
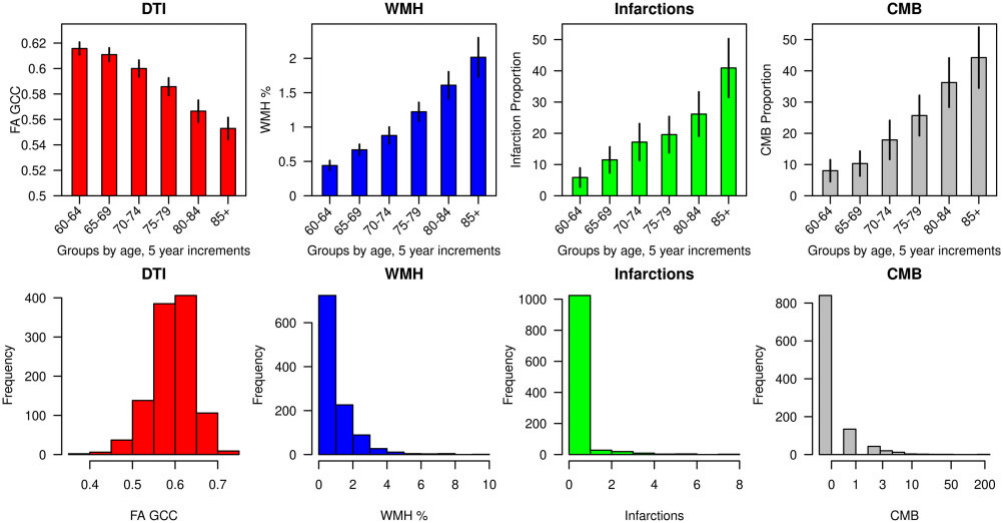
**Distributions of measured CVD (mean and two standard errors) and proportions by age**.

The overall participant characteristics as well as participants dichotomized by discovery and validation datasets are shown in Table 1. As expected, the participant characteristics were not different between the randomly sampled discovery and validation groups. The distributions (mean and two standard errors) and proportions by age of the measured CVD changes are shown in Fig. 2.

**Table 1:** Characteristics table by discovery and validation datasets with the mean (SD) listed for the continuous variables and count (%) for the categorical variables

	All *n* = 1089	Discovery *n* = 544	Validation *n* = 545	*P*-value
Demographics and subject characteristics				
Age, yrs	73.1 (8.4)	72.8 (8.4)	73.4 (8.4)	0.20
Male, no. (%)	587 (54%)	287 (53%)	300 (55%)	0.45
APOE4, no. (%)	318 (29%)	160 (30%)	158 (29%)	0.91
Education/Occupation	12.6 (2.6)	12.6 (2.6)	12.6 (2.6)	0.94
# of Cardiovascular metabolic conditions (CMC)	2.1 (1.5)	2.1 (1.5)	2.0 (1.4)	0.26
Imaging characteristics				
Amyloid	1.56 (0.38)	1.55 (0.38)	1.57 (0.38)	0.42
DTI (FA GCC)	0.59 (0.05)	0.59 (0.05)	0.60 (0.05)	0.19
WMH/TIV %	1.01 (1.04)	1.06 (1.12)	0.96 (0.95)	0.21
Infarction, no. (%)	191 (18%)	100 (18%)	91 (17%)	0.46
# of Infarctions	0.30 (0.86)	0.33 (0.90)	0.28 (0.81)	0.39
CMBs, no. (%)	217 (21%)	110 (21%)	107 (20%)	0.78
# of CMBs	0.68 (6.79)	0.97 (9.48)	0.39 (1.54)	0.16
Cognitive variables				
MMSE	28.2 (1.6)	28.2 (1.6)	28.2 (1.7)	0.91
Global *z*-scores	0.05 (1.10)	0.04 (1.10)	0.06 (1.11)	0.81
Memory *z*-scores	0.09 (1.14)	0.10 (1.11)	0.07 (1.17)	0.72
Attention *z*-scores	−0.08 (1.13)	−0.05 (1.13)	−0.10 (1.13)	0.52
Language *z*-scores	−0.06 (1.12)	−0.07 (1.10)	−0.05 (1.14)	0.72
Visual-spatial *z*-scores	0.12 (1.03)	0.08 (1.04)	0.16 (1.01)	0.24
MCI, no. (%)	104 (10%)	56 (10%)	48 (9%)	0.89
Follow-up time interval, yrs.	4.0 (1.6)	4.1 (1.7)	4.0 (1.6)	0.66

* *P*-values for differences between groups come from a *t*-test for the continuous variables or a chi-squared test for the categorical variables. Amyloid has been reported in the units of PIB SUVr.

### Step 1: components of variability across MRI markers of CVD

**Figure 3:**
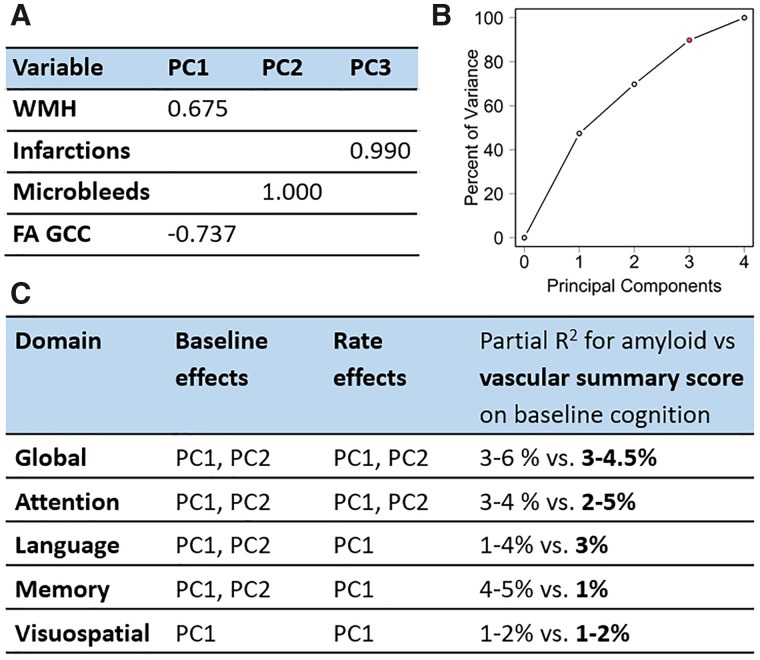
**Principal component analyses on discovery data**. (**A**) Components of CVD; (**B**) Percent of variance explained by each component; (**C**) Impact on baseline and rate of change in cognitive performance.

Using WMH, numbers of infarctions, number of CMBs, FA GCC measure as input in the discovery dataset, the PCA analyses yielded three main components as shown in Fig. 3. These components together explained 89.7% of the variability in the CVD Data. The first component (PC1) explained 45% of the variability and provided weightings after rotation of 0.675 for WMH and −0.737 for FA GCC. The second component (PC2) explained 25% of data variability and had a weighting after rotation of 1 for number of CMBs. The third component (PC3) explained 20% of the data variability and provided a weighting after rotation of 0.99 for number of infarctions.

### Step 2: prognostic value of the CVD components along with amyloidosis

The mixed effect models with longitudinal global *z*-score and domain specific *z*-scores as outcomes are shown in Table 2. We report results from both the discovery and validation datasets. Though there are subtle differences between the associations within the two groups, the coefficients were consistent between the two independent datasets. *Main effects on cognitive performance*: Older age, lower education/occupation and male sex were associated with lower baseline global and all domain *z*-scores (*P* < 0.05) except visuospatial *z*-scores where male sex was associated with better baseline *z*-scores. Higher amyloid, lower PC1, lower PC2, but not PC3 were associated with better baseline global and domain specific *z*-scores except for lack of association between PC2 and baseline visuospatial *z*-scores (*P* < 0.05). *Interaction with time effects on cognitive performance*: These are the predictors of future cognitive decline. Older age, higher amyloid load and lower PC1 were associated with greater rate of cognitive decline in all domains and global *z*-score (*P* < 0.05). On the other hand, PC2 was associated with the rate of decline for global and attention *z*-scores (*P* < 0.05). There were weak associations that were only found in the discovery dataset: male sex was associated with greater rate of decline of global and attention *z*-scores and higher education/occupation was associated with greater rate of decline in memory *z*-scores. The sensitivity analyses separately in A− and A+ individuals are shown in Supplementary Table 2. Comparing the magnitude of impact of the imaging biomarkers on cognitive decline for global cognitive decline over time, we found that amyloid had a greater magnitude of impact [Coefficient (standard error or SE, *P*-value): −0.21 (0.03, *P* < 0.001) for discovery and −0.21 (0.03, *P* < 0.001) for validation] in comparison to PC1 [Coefficient (SE, *P*-value): −0.02 (0.01, *P* < 0.001) for discovery and −0.01 (0.01, *P* = 0.037) for validation].

**Table 2:** The mixed effect models with each of the individual PCs of CVD as input and longitudinal global *z*-score and domain specific *z*-scores as outcomes. We have indicated amyloid and PC predictors in bold

Outcome	Predictor	Discovery		Validation	
		Coefficient (s.e.)	*P*-value	Coefficient (s.e.)	*P*-value
	Intercept	1.14 (0.48)	0.017	0.43 (0.48)	0.376
	Time	0.40 (0.06)	<0.001	0.48 (0.07)	<0.001
	Time^2^	−0.01 (0.002)	<0.001	−0.01 (0.002)	<0.001
Global *z*-scores	Age	−0.03 (0.01)	<0.001	−0.03 (0.01)	<0.001
	Male	−0.30 (0.08)	<0.001	−0.42 (0.08)	<0.001
	Educ/Occ	0.14 (0.02)	<0.001	0.17 (0.02)	<0.001
	**Amyloid**	−**1.09 (0.21)**	**<0.001**	−**0.79 (0.21)**	**<0.001**
	**PC1**	−**0.12 (0.04)**	**0.002**	−**0.10 (0.04)**	**0.011**
	**PC2**	−**0.08 (0.03)**	**0.013**	−**0.14 (0.04)**	**<0.001**
	Time*Age	−0.004 (0.001)	<0.001	−0.01 (0.001)	<0.001
	Time*Amyloid	−**0.21 (0.03)**	**<0.001**	−**0.21 (0.03)**	**<0.001**
	Time*PC1	−**0.02 (0.01)**	**<0.001**	−**0.01 (0.01)**	**0.037**
*R* ^2^ (fixed)		0.425		0.393	
	Intercept	1.92 (0.50)	<0.001	0.71 (0.48)	0.145
	Time	0.32 (0.08)	<0.001	0.45 (0.10)	<0.001
	Time^2^	−0.01 (0.003)	0.031	−0.01 (0.003)	0.001
Attention *z*-scores	Age	−0.04 (0.01)	<0.001	−0.03 (0.01)	<0.001
	Male	−0.41 (0.08)	<0.001	−0.42 (0.08)	<0.001
	Educ/Occ	0.11 (0.02)	<0.001	0.14 (0.02)	<0.001
	**Amyloid**	−**0.89 (0.21)**	**<0.001**	−**0.76 (0.21)**	**<0.001**
	**PC1**	−**0.15 (0.04)**	**<0.001**	−**0.15 (0.04)**	**<0.001**
	**PC2**	−**0.003 (0.03)**	**0.941**	−**0.13 (0.04)**	**<0.001**
	Time*Age	−0.004 (0.001)	0.001	−0.01 (0.001)	<0.001
	Time*Amyloid	−**0.14 (0.04)**	**<0.001**	−**0.18 (0.05)**	**<0.001**
	Time*PC1	−**0.03 (0.01)**	**<0.001**	−**0.01 (0.01)**	**0.212**
	Time*PC2	**0.02 (0.01)**	**0.048**	−**0.005 (0.01)**	**0.534**
*R* ^2^ (fixed)		0.399		0.372	
	Intercept	0.57 (0.49)	0.242	0.17 (0.52)	0.749
	Time	0.12 (0.02)	<0.001	0.08 (0.02)	<0.001
	Time^2^	−0.01 (0.003)	0.003	−0.003 (0.003)	0.216
Language *z*-scores	Age	−0.02 (0.01)	<0.001	−0.02 (0.01)	0.001
	Male	−0.34 (0.08)	<0.001	−0.47 (0.09)	<0.001
	Educ/Occ	0.13 (0.02)	<0.001	0.15 (0.02)	<0.001
	**Amyloid**	−**0.89 (0.21)**	**<0.001**	−**0.54 (0.22)**	**0.018**
	**PC1**	−**0.10 (0.04)**	**0.008**	−**0.04 (0.04)**	**0.387**
	**PC2**	−**0.14 (0.03)**	**<0.001**	−**0.16 (0.04)**	**<0.001**
	Time*Amyloid	−**0.27 (0.04)**	**<0.001**	−**0.27 (0.03)**	**<0.001**
	Time*PC1	−**0.02 (0.01)**	**<0.001**	−**0.03 (0.01)**	**<0.001**
*R* ^2^ (fixed)		0.336		0.300	
	Intercept	0.57 (0.52)	0.271	0.66 (0.53)	0.217
	Time	0.57 (0.09)	<0.001	0.54 (0.09)	<0.001
	Time^2^	−0.01 (0.003)	0.001	−0.01 (0.003)	0.004
Memory *z*-scores	Age	−0.02 (0.01)	0.007	−0.02 (0.01)	0.010
	Male	−0.53 (0.09)	<0.001	−0.65 (0.09)	<0.001
	Educ/Occ	0.12 (0.02)	<0.001	0.12 (0.02)	<0.001
	**Amyloid**	−**1.00 (0.22)**	**<0.001**	−**1.13 (0.23)**	**<0.001**
	**PC1**	−**0.10 (0.04)**	**0.022**	−**0.04 (0.04)**	**0.348**
	**PC2**	−**0.09 (0.04)**	**0.015**	−**0.08 (0.04)**	**0.036**
	Time*Age	−0.01 (0.001)	<0.001	−0.01 (0.001)	<0.001
	Time*Educ/Occ	−0.01 (0.003)	0.024	0.001 (0.003)	0.758
	Time*Amyloid	−**0.19 (0.04)**	**<0.001**	−**0.20 (0.04)**	**<0.001**
	Time*PC1	−**0.02 (0.01)**	**0.022**	−**0.004 (0.007)**	**0.578**
*R* ^2^ (fixed)		0.319		0.303	
	Intercept	0.21 (0.48)	0.668	−0.16 (0.46)	0.721
	Time	0.24 (0.07)	0.001	0.21 (0.07)	0.002
	Time^2^	−0.01 (0.003)	0.001	−0.01 (0.003)	0.009
Visuospatial *z*-scores	Age	−0.02 (0.01)	0.007	−0.02 (0.01)	0.001
	Male	0.23 (0.08)	0.003	0.10 (0.08)	0.200
	Educ/Occ	0.10 (0.01)	<0.001	0.14 (0.01)	<0.001
	**Amyloid**	−**0.61 (0.21)**	**0.004**	−**0.29 (0.20)**	**0.147**
	**PC1**	−**0.09 (0.04)**	**0.019**	−**0.10 (0.04)**	**0.009**
	Time*Age	−0.002 (0.001)	0.039	−0.002 (0.001)	0.031
	Time*Amyloid	−**0.14 (0.03)**	**<0.001**	−**0.16 (0.03)**	**<0.001**
	Time*PC1	−**0.02 (0.01)**	**<0.001**	−**0.02 (0.01)**	**0.009**
*R* ^2^ (fixed)		0.254		0.278	

### Step 3: prognostic value of a summary vascular score

**Figure 4:**
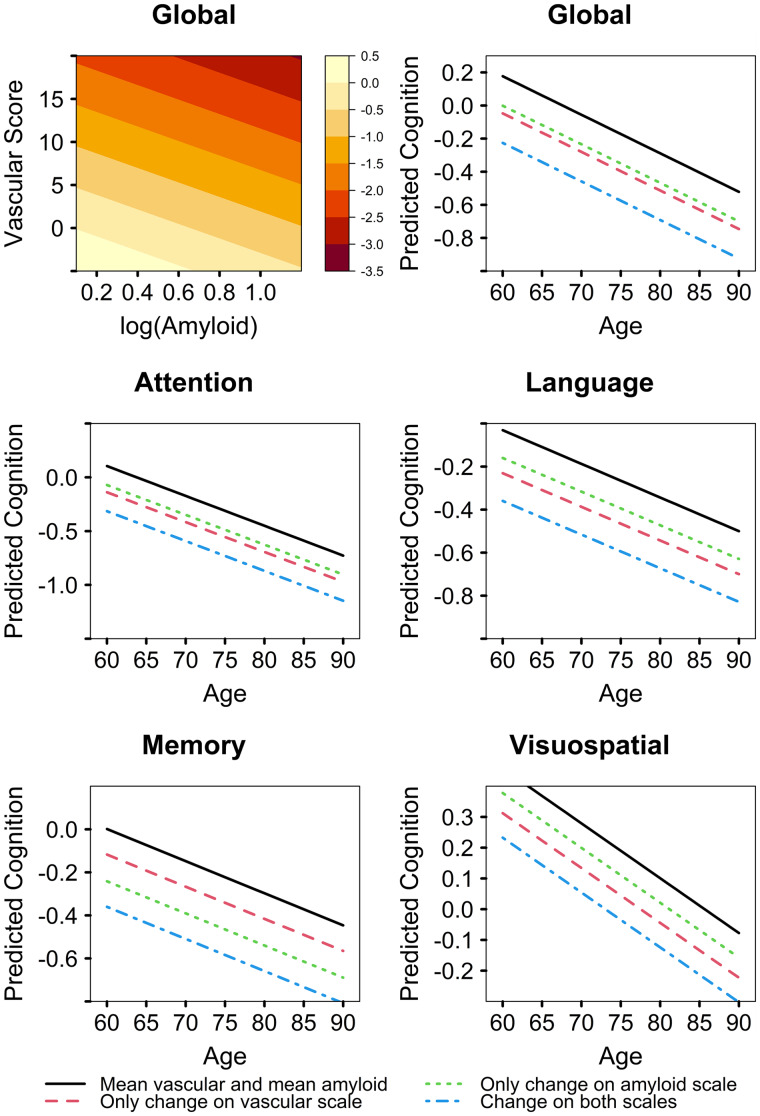
**Relative impact of vascular score and amyloidosis on baseline cognition (global *z*-scores and domain specific *z*-scores) as a function of age in the validation dataset**.

The linear regression model with vascular summary score as the predictor instead of the PCs is shown in Supplementary Table 1 for both the discovery and validation datasets. The contributions of age, sex and education/occupation were similar to Table 2. Higher amyloid and higher vascular score were predictors of lower baseline global and domain *z*-scores. We did not see any significant vascular score and amyloid interactions in either the discovery or validation data. The relative contributions (partial *R*^2^ values) of amyloid and vascular score for prediction of baseline cognition are also shown in the Supplementary Table 1 and summarized in Fig. 3. The vascular score explained 3–4.5% of variability in baseline global cognition in comparison to 3–6% variability explained by amyloidosis and had similar model *R*^2^ performance as individual PCs. Amyloid and vascular score had a similar impact on attention, language, and visuospatial *z*-scores which extended to the ranges provided in Fig. 3. However, the vascular score had the least impact on memory *z*-scores (∼1%) compared to an impact of 4–5% due to amyloidosis. These results can also be observed from the plots shown in Fig. 4 for the validation dataset and Supplementary Fig. 1 for the discovery dataset. The predicted cognition for the vascular summary score was lower than amyloid (lines are further apart) in Fig. 4 for memory in comparison to global *z*-scores and attention domains.

**Figure 5:**
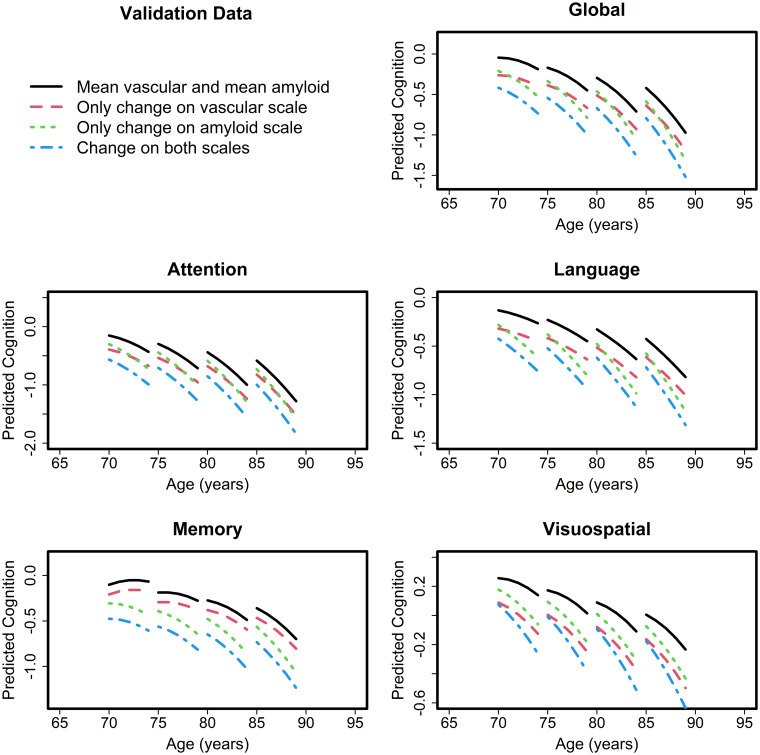
**Relative impact of vascular score and amyloidosis on longitudinal cognition (global *z*-scores and domain specific *z*-scores) as a function of age in the validation dataset**.

In the longitudinal models shown in Table 3, all the associations with baseline predictors were similar to those seen in Table 2 and described here. Higher age and amyloidosis were associated with faster rate of decline in global and domain *z*-scores (*P* < 0.05). However, the association between vascular score and rate of decline was only seen with visuospatial *z*-scores (*P* < 0.05) suggesting that the summation of PCs might have reduced the predictive ability of the CVD components. The longitudinal cognitive trajectories as a function of age are shown in Fig. 5 for the validation dataset and in Supplementary Fig. 2 for the discovery dataset.

**Table 3:** The mixed effect models with vascular score as the predictor instead of the components as input and longitudinal global *z*-score and domain specific *z*-scores as outcomes. We have indicated amyloid and vascular score predictors in bold

Outcome	Predictor	Discovery		Validation	
		Coefficient (s.e.)	*P*-value	Coefficient (s.e.)	*P*-value
	Intercept	1.26 (0.45)	0.005	0.38 (0.45)	0.407
	Time	0.56 (0.05)	<0.001	0.57 (0.05)	<0.001
	Time^2^	−0.01 (0.002)	<0.001	−0.01 (0.002)	<0.001
Global *z*-score	Age	−0.03 (0.01)	<0.001	−0.03 (0.01)	<0.001
	Male	−0.28 (0.08)	<0.001	−0.41 (0.08)	<0.001
	Educ/Occ	0.14 (0.02)	<0.001	0.17 (0.02)	<0.001
	**Amyloid**	−**1.15 (0.21)**	**<0.001**	−**0.81 (0.21)**	**<0.001**
	**Vascular score**	−**0.07 (0.02)**	**<0.001**	−**0.10 (0.02)**	**<0.001**
	Time*age	−0.01 (0.001)	<0.001	−0.01 (0.001)	<0.001
	Time*amyloid	−**0.21 (0.03)**	**<0.001**	−**0.22 (0.03)**	**<0.001**
*R* ^2^ (fixed)		0.415		0.391	
	Intercept	2.31 (0.47)	<0.001	0.83 (0.45)	0.069
	Time	0.49 (0.07)	<0.001	0.53 (0.08)	<0.001
	Time^2^	−0.01 (0.003)	0.031	−0.01 (0.003)	0.002
Attention *z*-score	Age	−0.04 (0.01)	<0.001	−0.03 (0.01)	<0.001
	Male	−0.36 (0.08)	<0.001	−0.39 (0.08)	<0.001
	Educ/Occ	0.11 (0.02)	<0.001	0.14 (0.02)	<0.001
	**Amyloid**	−**0.92 (0.22)**	**<0.001**	−**0.79 (0.21)**	**<0.001**
	**Vascular score**	−**0.06 (0.02)**	**0.004**	−**0.11 (0.02)**	**<0.001**
	Time*age	−0.01 (0.001)	<0.001	−0.01 (0.001)	<0.001
	Time*amyloid	−**0.14 (0.04)**	**0.001**	−**0.18 (0.05)**	**<0.001**
*R* ^2^ (fixed)		0.383		0.369	
	Intercept	2.38 (0.42)	<0.001	1.95 (0.45)	<0.001
	Time	0.37 (0.07)	<0.001	0.39 (0.06)	<0.001
	Time^2^	−0.01 (0.003)	0.002	−0.003 (0.003)	0.214
Language *z*-score	Age	−0.03 (0.01)	<0.001	−0.02 (0.01)	0.002
	Male	−0.21 (0.08)	0.012	−0.35 (0.09)	<0.001
	**Amyloid**	−**1.00 (0.23)**	**<0.001**	−**0.66 (0.24)**	**0.006**
	**Vascular score**	−**0.09 (0.02)**	**<0.001**	−**0.08 (0.02)**	**<0.001**
	Time*age	−0.004 (0.001)	<0.001	−0.004 (0.001)	<0.001
	Time*amyloid	−**0.25 (0.04)**	**<0.001**	−**0.25 (0.04)**	**<0.001**
*R* ^2^ (fixed)		0.263		0.193	
	Intercept	0.61 (0.49)	0.207	0.55 (0.50)	0.267
	Time	0.66 (0.08)	<0.001	0.55 (0.08)	<0.001
	Time^2^	−0.01 (0.003)	0.001	−0.01 (0.003)	0.004
Memory *z*-score	Age	−0.02 (0.01)	0.003	−0.02 (0.01)	0.011
	Male	−0.51 (0.08)	<0.001	−0.65 (0.09)	<0.001
	Educ/Occ	0.12 (0.02)	<0.001	0.12 (0.02)	<0.001
	**Amyloid**	−**1.06 (0.22)**	**<0.001**	−**1.14 (0.23)**	**<0.001**
	**Vascular score**	−**0.07 (0.02)**	**0.001**	−**0.05 (0.02)**	**0.013**
	Time*age	−0.01 (0.001)	<0.001	−0.01 (0.001)	<0.001
	Time*educ/occ	−0.01 (0.003)	0.032	0.001 (0.003)	0.696
	Time*amyloid	−**0.19 (0.04)**	**<0.001**	−**0.20 (0.04)**	**<0.001**
*R* ^2^ (fixed)		0.314		0.303	
	Intercept	0.23 (0.46)	0.616	−0.35 (0.43)	0.424
	Time	0.30 (0.07)	<0.001	0.20 (0.06)	0.002
	Time^2^	−0.01 (0.003)	0.006	−0.01 (0.003)	0.019
Visuospatial *z*-scores	Age	−0.02 (0.01)	0.006	−0.02 (0.01)	0.001
	Male	0.24 (0.08)	0.002	0.13 (0.08)	0.080
	Educ/Occ	0.10 (0.02)	<0.001	0.15 (0.01)	<0.001
	**Amyloid**	−**0.72 (0.21)**	**0.001**	−**0.27 (0.20)**	**0.171**
	**Vascular score**	−**0.04 (0.02)**	**0.032**	−**0.07 (0.02)**	**<0.001**
	Time*age	−0.003 (0.001)	0.028	−0.002 (0.001)	0.024
	Time*amyloid	−**0.15 (0.03)**	**<0.001**	−**0.15 (0.03)**	**<0.001**
	Time*vascular Score	−**0.01 (0.004)**	**0.016**	−**0.01 (0.003)**	**0.001**
*R* ^2^ (fixed)		0.245		0.292	

One may argue that it would be enough to measure only WMH as reflective of WM damage. Therefore, we also conducted sensitivity analyses with WMH alone versus PC1 (composite of WMH and FA GCC) as a part of the vascular composite score. We found that the partial *R*^2^ of the vascular score was slightly higher with PC1 when compared to WMH alone for prediction of global cognition. We also evaluated models predicting vascular summary scores with age, sex and systemic vascular health (measured using CMC or Cardiovascular metabolic conditions composite) in the entire cohort. We found that CMC was associated with the vascular summary score when FA GCC was included in the model (model *R*^2^ of 0.235, CMC *P* = 0.007) but was not associated with the vascular summary score formed with only WMH as a component (model *R*^2^ of 0.076, CMC *P* = 0.71). These results lend support for inclusion of dMRI based measures in CVD scores because these changes aid in capturing early vascular risk related brain changes.

## Discussion

Dementia is most commonly a multi-factorial process particularly in elderly persons wherein multiple, coexisting brain pathologies result in progressive cognitive impairment which ultimately leads to dementia. Pathologic studies have shown that CVD is common among elderly and it lowers the threshold of clinically overt dementia in the presence of other neurodegenerative pathologies.^34^ Although there is an impetus to standardize criteria for clinical diagnosis, pathological diagnosis, and neuroimaging image interpretation and reporting in vascular cognitive impairment^3^^,^^35–37^ including large initiatives such as NIH lead MarkVCID, there is no optimized methodology that is available for the inclusion of CVD information in clinical trials for Alzheimer’s disease and other dementias. The major challenge has been that CVD is heterogeneous and one measure or image cannot sufficiently capture all the variability.

In this work, we found that MRI based CVD measurements could be divided into three sources of variability in a population-based sample. WM changes (PC1) represent a dynamic ongoing process measured using WMH and FA GCC and has a significant impact on rate of cognitive decline. Lobar and deep CMBs (PC2) measured on T_2_* GRE represents small vessel disease and had a significant impact on baseline cognitive performance and longitudinal change in the attention domain *z*-scores. Infarctions (PC3) (cortical and subcortical) measured on 2D FLAIR MRI have lower impact on cognitive trajectories in comparison to the effect of WM changes and CMBs on cognitive performance. All these three sources of CVD measurements taken together from MRI have a measurable impact on cognitive trajectories which is comparable to the impact of amyloidosis except for the memory domain.

### White matter changes due to CVD

WM changes were the primary source of variability that we observed across all MRI based CVD measurements. There are multiple mechanisms for WM changes due to CVD including hypoperfusion, altered water content, blood–brain barrier dysfunction, myelin damage and axonal disruption.^38^ WMH are the most studied CVD-related MRI changes that are largely vascular in origin. In the recent past, there has been a distinction made between subcortical and periventricular WMH due to blood supply differences to these two regions. The suggestion has been made that subcortical WMH are correlated with hypertension whereas periventricular changes driven by neuroinflammation response to blood–brain barrier dysfunction and possibly cerebral amyloid angiopathy.^38^ However, there is no clear demarcation between the mechanisms and regional propensity of WMH and deep and periventricular WMH are highly correlated with each other,^30^ therefore, we decided to utilize a single measure of global WMH as our WMH measure in the PCA analyses.

dMRI provides us with measures of WM microstructural integrity. Changes even in normal appearing WM associated with myelin damage, axonal loss and gliosis are expected and have been suggested to be captured using dMRI and these changes are predictive of cognitive performance.^9^^,^^10^ The pathologic basis of the diffusion changes needs further confirmation. However, there is strong evidence that diffusion related changes in the tissue microstructure are reflective of progressive WM damage even before the appearance of WMH.^6^^,^^7^ In a recent study, we found that the microstructural changes to the frontal WM, specifically genu or anterior corpus callosum, correlated well with vascular risk even in the absence of visible CVD. Since there are greater sclerotic changes of the frontal lobe medullary arteries due to hypertension^39^ and greater vulnerability of the frontal lobes to myelin loss and degeneration with vascular dementia compared to other neurodegenerative dementias,^40^^,^^41^ it would make sense that diffusion measurements in the frontal lobes were identified as good surrogates of vascular risk related damage. In addition, using the anterior portion of the largest interhemispheric WM bundle i.e. genu of the corpus callosum which has no crossing fibres lends itself to imaging measures that have low measurement variability.

Given that WMH and FA GCC both reflect WM damage and are highly correlated (*r* = −0.569), it is logical that these two measures were grouped together as PC1. In addition, WM changes represent a dynamic process in contrast to CMBs and infarcts that are discrete events which may cause a step-down in brain health and cognition with the appearance of each lesion. WM changes also capture the secondary degeneration due to the appearance of lesions which is the possible reason behind the significant contribution towards baseline cognitive performance and cognitive decline over time. The sensitivity analyses we performed showed that including dMRI along with WMH improved the model prediction of cognition and helped capture systemic vascular health related changes. Therefore, including both measures provides a more complete picture of WM health. In this study, we found that WMH and dMRI changes together have significant impact on cognition, and specifically on attention consistent with the literature (DeCarli et al., 2005b; Kantarci et al., 2011; Croall et al., 2017). Therefore, capturing these dynamic WM processes will be important to account for the impact of CVD on prognosis in ageing and dementia studies.

### Cerebral microbleeds and infarctions

CMBs are common in the elderly and are associated with cognitive performance.^20^^,^^42^^,^^43^ CMBs are small haemorrhages likely due to the structural weakening and rupture of the small vessels.^44^ The mechanisms of deep versus lobar CMBs are suggested to be distinct with stronger association between deep CMBs and hypertension in comparison to the stronger association between lobar CMBs and cerebral amyloid angiopathy.^42^^,^^45^ We found that the number of CMBs (either lobar or deep) captured the extent of small vessel disease in individuals and were grouped together in the PCA analyses. Such a grouping irrespective of location and number made sense because the presence of CMBs reflects the vulnerability of small vessels to CMBs and is therefore the simplest to use. We found that the number of CMBs had a significant impact on global cognitive performance which is consistent with the literature^42^^,^^43^ and the impact was larger on the attention domain. We acknowledge that measurement of CMBs on susceptibility weighted imaging (SWI) may be more sensitive in capturing small vessel changes but we were limited to using T_2_* GRE in this study due to its availability over the last decade. Future studies will be able to evaluate the associations between CMBs and cognition with more sensitive markers. Others have summed CMBs, WMH, enlarged perivascular spaces and lacunar infarcts in a cerebral small vessel disease metric.^46^ While subcortical infarcts, WMH and CMBs might have been grouped together since they are manifestations of cerebral small vessel disease, they did not in our analysis.

Infarctions are the least common of the PCs measured in this study with a prevalence of about 10% in the population.^20^^,^^47^ Infarctions are considered to be discrete events which cause a step down in cognitive performance. Therefore, we expected that the impact of PC3 would be on baseline cognition and not rate of cognitive decline. However, we did not find evidence for an association between infarctions and cognitive performance. There could be four possible reasons behind the lack of association: (i) since a lower percentage of participants have infarctions, we did not have sufficient sample size to detect the association; (ii) WM changes (secondary to the infarctions) sufficiently captured the variability in cognition; (iii) a large proportion of those with infarctions have silent infarctions (>85%) which suggests that the impact on cognition maybe minimal; and (iv) there is significant variability in the location of the infarctions so taken together their composite effect on any specific domain score is low.

### Composite scores for CVD: Good versus Bad

Even though we could have evaluated all of the measures obtained from FLAIR, T_2_* GRE and FA GCC separately in models, the PCA analyses allowed us to summarize the data along axes that captured the most variance to reduce the number of dimensions and to understand the heterogeneity which is helpful. While we had expected greater variability in the way PC axes were formed especially when we had originally considered regional information in our analyses, we were surprised to find that three simple axes explained most of the variability in the CVD measures from these three images. Simply put, our results suggest that individuals are more similar in terms of their WM changes (PC1), CMBs (PC2) and infarctions (PC3) irrespective of their location. At the basic level, these axes are likely capturing the vulnerability of each individual to each of these processes. A question arises if these PCs should be further summarized.

In the past decade, there have been a few attempts to enable neuropathological and imaging-based quantification of CVD^48–52^ where each component was either given equal weight or weighted based on its importance for prediction of cognitive performance. In this work, we tested a simple approach of summing up the individual’s PCs because relying heavily on the relationship between CVD measures and cognitive performance in the discovery dataset to compute weights can limit the generalizability of the score to other datasets.

The advantage of a single CVD measure is that it makes it easy to account for CVD in studies and also understand the contribution of CVD in comparison to other factors (as in Table 3). However, composite measures make it difficult to understand mechanisms and will have reduced prognostic ability in comparison to the individual PCs independently because we are averaging all the components which clearly have varied prognostic ability. This can be observed in Table 3 where the vascular score only influenced the rate of decline in visuospatial *z*-scores whereas PC1 was a predictor of rate of decline seen in all domains (in Table 2). Therefore, it may be advantageous to measure WM changes (PC1) and CMBs (PC2) independently in ageing and dementia studies to account for CVD.

### Amyloidosis, cerebrovascular disease and cognitive trajectories

Alzheimer’s disease and CVD are two major components of cognitive impairment in the population. This is reflected by the relative contribution of amyloidosis versus CVD in Table 3 with the main difference being that amyloidosis had a greater impact on memory domain in comparison to CVD measures which is widely accepted. While there is literature on some overlap between Alzheimer’s disease and CVD mechanisms, there has been no strong evidence for the interaction of both mechanisms.^53^ Furthermore, our work has shown that CVD has greater predilection for WM changes in comparison to Alzheimer’s disease.^54^ This literature supports the lack of amyloid and vascular score associations in Table 3 and Supplementary Table 1. As a sensitivity analysis, we also conducted analyses in A− and A+ participants separately and found similar PCs results (extent of effect of each feature and PCs on cognition) in the linear regressions models, suggesting that the associations between the vascular scores and cognition were generally not different between the amyloid negative and positive groups. While these results were seen in a population-based sample, this does not preclude the possibility that the extent of CVD and impact on cognition may be very different and possibly lower in dementia individuals.^55^

### Strengths, limitations and future work

The strengths of the study include a data driven evaluation of the mechanisms underlying CVD measures that are commonly measured in ageing and dementia studies. Drawbacks include the fact that the CVD measurements available in our data set were not exhaustive and may have limited sensitivity and specificity. For example, we did not include measures of CBF or perivascular spaces, although measurements of perivascular spaces are not highly reproducible and impact on cognitive performance needs further confirmation.^5^ We also had limited power to detect subtle differences between uncommon CVD measures. For example, differences between deep microbleeds (present in 26 individuals in the discovery data) and lobar microbleeds (present in 96) would be of interest but might require a larger or enriched sample. In the future, we will pursue advanced dMRI techniques such as Neurite orientation dispersion and density imaging and multi compartment Free Water Elimination to quantify intra and extracellular microscopic features of WM. We will also utilize SWI and 3D FLAIR assessments for the sensitive measurement of CVD.

## Supplementary material

Supplementary material is available at *Brain Communications* online.

## Supplementary Material

fcab076_Supplementary_Data

## Abbreviations

CMB =: cerebral microbleed
CVD =: cerebrovascular disease
dMRI =: diffusion MRI
FA GCC =: genu of the corpus callosum fractional anisotropy
FLAIR =: fluid-attenuated inversion recovery
PC =: principal component
PCA =: principal component analyses
T_2_*-GRE =: T_2_*-weighted gradient-echo (GRE)
WM =: white matter
WMH s =: white matter hyperintensities
