# Supplementary material for: White matter abnormalities are key components of cerebrovascular disease impacting cognitive decline
Source: Brain Commun. 2021 Apr 12;3(2):fcab076. doi: 10.1093/braincomms/fcab076 (PMC8072521; doi:10.1093/braincomms/fcab076)
Supplement: fcab076_Supplementary_Data [file fcab076_supplementary_data.docx]

**Supplemental Table 1.** Linear regression model with vascular score as the predictor and baseline cognition as an outcome. Partial R^2^ for Vascular Score in (), and partial R^2^ for amyloid in []

| Outcome | Predictor |  | Discovery (n=544) |  |  | Validation (n=545) |  |
| --- | --- | --- | --- | --- | --- | --- | --- |
|  |  | R^2^ | Coefficient (s.e.) | P-value | R^2^ | Coefficient (s.e.) | P-value |
|  |  | **0.378** |  |  | **0.342** |  |  |
|  | Intercept |  | 1.15 (0.45) | 0.011 |  | 0.31 (0.46) | 0.503 |
| Global  z-scores | Age |  | -0.03 (0.01 | <0.001 |  | -0.02 (0.01) | <0.001 |
|  | Male |  | -0.28 (0.08) | <0.001 |  | -0.41 (0.08) | <0.001 |
|  | Educ/Occ |  | 0.14 (0.02) | <0.001 |  | 0.16 (0.02) | <0.001 |
|  | Amyloid | [0.063] | -1.20 (0.21) | <0.001 | [0.031] | -0.87 (0.22) | 0.001 |
|  | Vascular Score | (0.029) | -0.08 (0.02) | <0.001 | (0.045) | -0.10 (0.02) | <0.001 |
|  |  |  |  |  |  |  |  |
|  |  | **0.367** |  |  | **0.305** |  |  |
|  | Intercept |  | 2.05 (0.46) | <0.001 |  | 0.92 (0.48) | 0.057 |
| Attention  z-scores | Age |  | -0.04 (0.01) | <0.001 |  | -0.03 (0.01) | <0.001 |
|  | Male |  | -0.34 (0.08) | <0.001 |  | -0.36 (0.09) | <0.001 |
|  | Educ/Occ |  | 0.11 (0.02) | <0.001 |  | 0.12 (0.02) | <0.001 |
|  | Amyloid | [0.041] | -0.99 (0.21) | <0.001 | [0.028] | -0.86 (0.22) | <0.001 |
|  | Vascular Score | (0.020) | -0.07 (0.02) | 0.001 | (0.049) | -0.11 (0.02) | <0.001 |
|  |  |  |  |  |  |  |  |
|  |  | **0.287** |  |  | **0.243** |  |  |
|  | Intercept |  | 0.33 (0.48) | 0.492 |  | -0.23 (0.50) | 0.641 |
| Language  z-scores | Age |  | -0.02 (0.01) | 0.002 |  | -0.02 (0.01) | 0.013 |
|  | Male |  | -0.32 (0.08) | <0.001 |  | -0.45 (0.09) | <0.001 |
|  | Educ/Occ |  | 0.13 (0.02) | <0.001 |  | 0.15 (0.02) | <0.001 |
|  | Amyloid | [0.042] | -1.06 (0.22) | <0.001 | [0.014] | -0.63 (0.23) | 0.006 |
|  | Vascular Score | (0.032) | -0.09 (0.02) | <0.001 | (0.030) | -0.09 (0.02) | <0.001 |
|  |  |  |  |  |  |  |  |
|  |  | **0.253** |  |  | **0.249** |  |  |
|  | Intercept |  | 0.76 (0.50) | 0.130 |  | 0.47 (0.51) | 0.358 |
| Memory  z-scores | Age |  | -0.02 (0.01) | 0.002 |  | -0.01 (0.01) | 0.019 |
|  | Male |  | -0.51 (0.09) | <0.001 |  | -0.67 (0.09) | <0.001 |
|  | Educ/Occ |  | 0.12 (0.02) | <0.001 |  | 0.13 (0.02) | <0.001 |
|  | Amyloid | [0.043] | -1.09 (0.23) | <0.001 | [0.047] | -1.19 (0.23) | <0.001 |
|  | Vascular Score | (0.009) | -0.05 (0.02) | 0.032 | (0.011) | -0.05 (0.02) | 0.019 |
|  |  |  |  |  |  |  |  |
|  |  | **0.176** |  |  | **0.241** |  |  |
|  | Intercept |  | 0.07 (0.48) | 0.883 |  | -0.19 (0.45) | 0.670 |
| Visuospatial  z-scores | Age |  | -0.01 (0.01) | 0.018 |  | -0.02 (0.01) | 0.002 |
|  | Male |  | 0.26 (0.08) | 0.002 |  | 0.15 (0.08) | 0.057 |
|  | Educ/Occ |  | 0.10 (0.02) | <0.001 |  | 0.14 (0.02) | <0.001 |
|  | Amyloid | [0.023] | -0.77 (0.22) | <0.001 | [0.007] | -0.39 (0.21) | 0.067 |
|  | Vascular Score | (0.010) | -0.05 (0.02) | 0.023 | (0.020) | -0.07 (0.02) | 0.002 |

**Supplemental Table 2:** Within the amyloid negative (A-) and amyloid positive groups (A+), CVD features as predictors in the regression models as well as PCs based on A- with cognitive performance as an outcome. While the different n in each group are reflected by different p-values, the coefficients are similar for each predictor in the A- and A+ subgroups.

| Outcome | Predictor |  | A- (n=710) |  |  | A+ (n=382) |  |
| --- | --- | --- | --- | --- | --- | --- | --- |
|  |  | Model R^2^ | Coefficient (s.e.) | P-value | Model R^2^ | Coefficient (s.e.) | P-value |
| **CVD features as predictors** | | | | | | | |
| Global z-scores | WMH | 0.111 | -0.15 (0.04) | <0.001 | 0.112 | -0.32 (0.08) | <0.001 |
|  | Infarctions |  | -0.10 (0.04) | 0.013 |  | 0.03 (0.07) | 0.654 |
|  | CMB (Number) |  | -0.09 (0.03) | 0.008 |  | -0.12 (0.06) | 0.058 |
|  | FA GCC |  | 0.13 (0.04) | 0.001 |  | 0.10 (0.08) | 0.189 |
|  |  |  |  |  |  |  |  |
| Attention z-scores | WMH | 0.132 | -0.13 (0.04) | 0.002 | 0.124 | -0.32 (0.08) | <0.001 |
|  | Infarctions |  | -0.10 (0.04) | 0.013 |  | -0.02 (0.06) | 0.710 |
|  | CMB (Number) |  | -0.09 (0.03) | 0.012 |  | -0.01 (0.06) | 0.849 |
|  | FA GCC |  | 0.20 (0.04) | <0.001 |  | 0.17 (0.08) | 0.032 |
|  |  |  |  |  |  |  |  |
| Language z-scores | WMH | 0.082 | -0.17 (0.05) | <0.001 | 0.084 | -0.25 (0.08) | 0.001 |
|  | Infarctions |  | -0.06 (0.04) | 0.138 |  | 0.01 (0.06) | 0.864 |
|  | CMB (Number) |  | -0.14 (0.04) | <0.001 |  | -0.21 (0.06) | 0.001 |
|  | FA GCC |  | 0.05 (0.04) | 0.308 |  | 0.05 (0.08) | 0.538 |
|  |  |  |  |  |  |  |  |
| Memory z-scores | WMH | 0.034 | -0.12 (0.05) | 0.009 | 0.081 | -0.34 (0.08) | <0.001 |
|  | Infarctions |  | -0.09 (0.04) | 0.038 |  | 0.05 (0.07) | 0.425 |
|  | CMB (Number) |  | -0.06 (0.04) | 0.110 |  | -0.13 (0.07) | 0.056 |
|  | FA GCC |  | -0.02 (0.04 | 0.727 |  | 0.01 (0.08) | 0.935 |
|  |  |  |  |  |  |  |  |
| Visuospatial z-scores | WMH | 0.066 | -0.09 (0.04) | 0.051 | 0.042 | -0.21 (0.07) | 0.003 |
|  | Infarctions |  | -0.07 (0.04) | 0.080 |  | 0.04 (0.06) | 0.506 |
|  | CMB (Number) |  | -0.03 (0.04) | 0.478 |  | -0.05 (0.06) | 0.384 |
|  | FA GCC |  | 0.16 (0.04) | <0.001 |  | 0.01 (0.07) | 0.852 |
| **PCs as predictors** | | | | | | | |
| Global z-scores | PC1 | 0.111 | 0.20 (0.03) | <0.001 | 0.103 | 0.30 (0.05) | <0.001 |
|  | PC2 |  | 0.09 (0.03) | 0.007 |  | 0.11 (0.06) | 0.076 |
|  | PC3 |  | 0.10 (0.04) | 0.011 |  | -0.03 (0.07) | 0.605 |
|  |  |  |  |  |  |  |  |
| Attention z-scores | PC1 | 0.131 | 0.23 (0.03) | <0.001 | 0.120 | 0.34 (0.05) | <0.001 |
|  | PC2 |  | 0.09 (0.03) | 0.013 |  | 0.01 (0.06) | 0.914 |
|  | PC3 |  | 0.09 (0.04) | 0.015 |  | 0.02 (0.07) | 0.740 |
|  |  |  |  |  |  |  |  |
| Language z-scores | PC1 | 0.077 | 0.15 (0.03) | <0.001 | 0.077 | 0.21 (0.05) | <0.001 |
|  | PC2 |  | 0.14 (0.04) | <0.001 |  | 0.21 (0.06) | 0.001 |
|  | PC3 |  | 0.07 (0.04) | 0.086 |  | -0.01 (0.07) | 0.823 |
|  |  |  |  |  |  |  |  |
| Memory z-scores | PC1 | 0.029 | 0.07 (0.03) | 0.029 | 0.064 | 0.24 (0.06) | <0.001 |
|  | PC2 |  | 0.06 (0.04) | 0.089 |  | 0.12 (0.07) | 0.072 |
|  | PC3 |  | 0.10 (0.04) | 0.020 |  | -0.06 (0.07) | 0.381 |
|  |  |  |  |  |  |  |  |
| Visuospatial z-scores | PC1 | 0.065 | 0.17 (0.03) | <0.001 | 0.033 | 0.15 (0.05) | 0.001 |
|  | PC2 |  | 0.02 (0.04) | 0.502 |  | 0.04 (0.06) | 0.425 |
|  | PC3 |  | 0.07 (0.04) | 0.093 |  | -0.04 (0.06) | 0.456 |


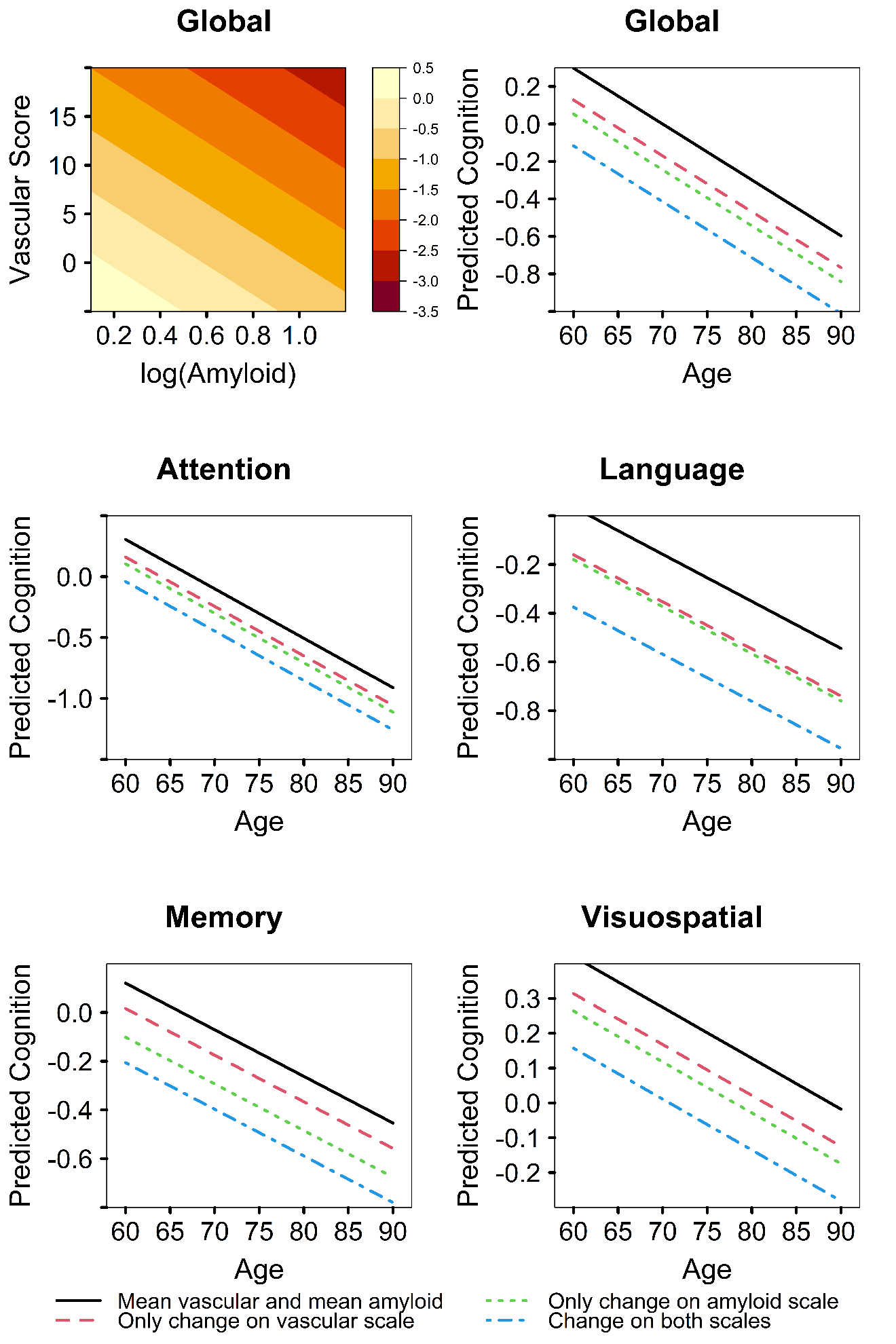


Supplementary Figure 1: Relative impact of vascular score and amyloidosis on baseline cognition (global z-scores and domain specific z-scores) as a function of age in the discovery dataset.


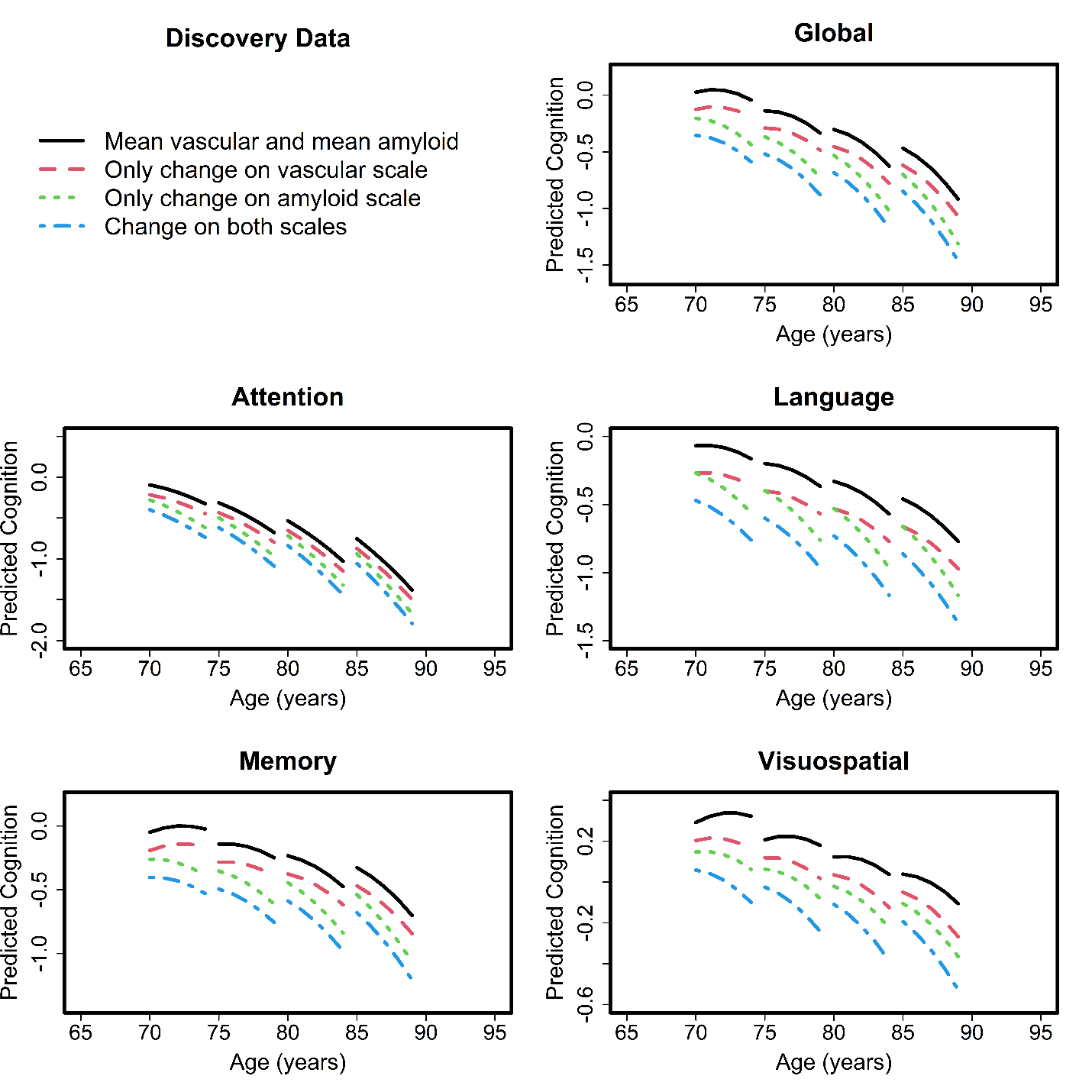


Supplementary Figure 2: Relative impact of vascular score and amyloidosis on longitudinal cognition (global z-scores and domain specific z-scores) as a function of age in the discovery dataset.
